# Multiferroic oxide BFCNT/BFCO heterojunction black silicon photovoltaic devices

**DOI:** 10.1038/s41377-021-00644-0

**Published:** 2021-09-26

**Authors:** Kaixin Guo, Xu Wang, Rongfen Zhang, Zhao Fu, Liangyu Zhang, Guobin Ma, Chaoyong Deng

**Affiliations:** 1 Key Laboratory of Electronic Composites of Guizhou Province, College of Big Data and Information Engineering, Guizhou University, Guiyang, 550025 Guizhou China; 2 Guizhou College of Electronic Science and Technology, Guiyang, 561113 Guizhou China

**Keywords:** Silicon photonics, Optoelectronic devices and components

## Abstract

Multiferroics are being studied increasingly in applications of photovoltaic devices for the carrier separation driven by polarization and magnetization. In this work, textured black silicon photovoltaic devices are fabricated with Bi_6_Fe_1.6_Co_0.2_Ni_0.2_Ti_3_O_18_/Bi_2_FeCrO_6_ (BFCNT/BFCO) multiferroic heterojunction as an absorber and graphene as an anode. The structural and optical analyses showed that the bandgap of Aurivillius-typed BFCNT and double perovskite BFCO are 1.62 ± 0.04 eV and 1.74 ± 0.04 eV respectively, meeting the requirements for the active layer in solar cells. Under the simulated AM 1.5 G illumination, the black silicon photovoltaic devices delivered a photoconversion efficiency (*η*) of 3.9% with open-circuit voltage (*V*_*oc*_), short-circuit current density (*J*_*sc*_), and fill factor (*FF*) of 0.75 V, 10.8 mA cm^−2^, and 48.3%, respectively. Analyses of modulation of an applied electric and magnetic field on the photovoltaic properties revealed that both polarization and magnetization of multiferroics play an important role in tuning the built-in electric field and the transport mechanisms of charge carriers, thus providing a new idea for the design of future high-performance multiferroic oxide photovoltaic devices.

## Abstract (web summary)

Regulation of photovoltaic response in multiferroic oxide BFCNT/BFCO heterojunction black silicon photovoltaic devices by polarization and magnetization.

## Graphical abstract

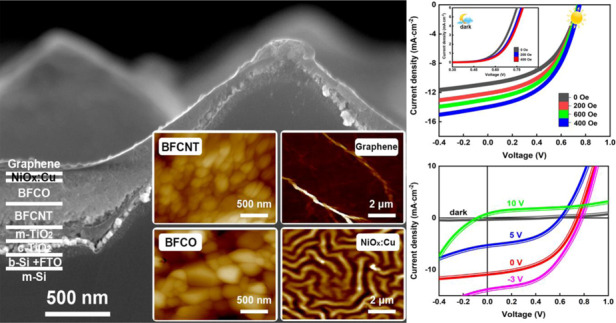

## Introduction

Multiferroic materials have been widely used as alternatives for photoelectric devices, such as photodiodes, solar cells, and photo field-effect transistors, etc.^1–5^. The low reversal symmetry state with spontaneous polarization induced by ferroelectric dipoles^6^ introduces shift current and allows the generation of a photo-generated voltage higher than the optical bandgap^7^. Since the discovery of the photovoltaic effect in bismuth ferrite (BiFeO_3_, BFO), solar cells based on ferroelectrics have been widely studied for their unique advantages, such as higher photovoltage, better photoelectric conversion and distinct regulation of the electric field, *etc*^8–10^. In these materials, the electron-electron interaction regulating magnetic ordering induces a smaller bandgap (*E*_*g*_), e.g. the *E*_*g*_ of BFO lies in the range of 2.6–2.7 eV, but 1.4–2.4 eV for double-perovskite Bi_2_FeCrO_6_ (BFCO). For Aurivillius (AU) compound Bi_6_Fe_2−*x*_Co_*x*/2_Ni_*x*/2_Ti_3_O_18_ (0 ≤ x ≤ 1), *E*_*g*_ varies with *x*, especially when *x* = 0.4 eV (Bi_6_Fe_1.6_Co_0.2_Ni_0.2_Ti_3_O_18_, BFCNT), the *E*_*g*_ can be as small as 1.58 eV, etc.^11–13^. Although the efficiency of these solar cells is still low owing to the poor overall conduction caused by their large bandgap and intrinsic dielectric properties, these ferroelectric photovoltaic devices empower with the potential to exceed the *Shockley*-*Queisser* limit, theoreticlly^14^.

Certainly, the increase of efficiency in such solar cells first requires the bandgap of these ferroelectrics to lie between 1.1 and 1.9 eV depend mainly on the material itself, a small bandgap brings not only more nonradiative transitions of carriers, causing a larger loss of the absorbed sunlight, but a small open-circuit voltage (*V*_*oc*_, V)^13^. However, a large bandgap causes less absorption of light. To compensate for the trade-off of the absorption, short circuit current (*I*_*sc*_, A), and *V*_*oc*_, the optimal *E*_*g*_ is 1.5 eV for single-junction solar cells^15^. Meanwhile, an adjustment of device structure can further improve the efficiency, the most common methods include reducing resistance between the cell and wiring, changing the electrode geometry, improving the phototonus, introducing an additional anti-reflection layer, roughening the surface, connecting two or more cells of different bandgaps, etc.^16–20^. For silicon-based solar cells, it is also important to texture the surface, which can increase not only the surface area but light absorption. Aided by selective absorption to the solar spectrum of tandem cells with different bandgaps, the efficiency can be enhanced prominently, as is reported recently by Ashouri, et al. that the efficiency of a tandem solar cell of monolithic perovskite/silicon reaches 29.15% by enhancing hole extraction rate, with an *E*_*g*_ of 1.68 eV^21^.

It is worth noting that the polarization in these ferroelectric perovskite solar cells plays a leading role in the separation and transport of photo-excited carriers^21–23^, the mechanism of which differs from the charge separation in *Schottky* and p–n diode photovoltaic devices, determined largely by the built-in electric fields^24,25^. Solar photovoltaic devices based on ferroelectrics changes reversibly the directions for the photo-generated current or photovoltage by regulating the polarization direction and may also form a large photovoltage exceeding bandgaps of materials^26^. However, there are only a few pieces of research on the effect of magnetization on photovoltaic characteristics^27^.

Herein, we report, for the first time, a graphene-based multiferroic oxide BFCNT/BFCO heterojunction black silicon (b-Si) solar cell. First, the optical and ferroelectric characterization of non-toxic muliferroic bismuth layered perovskites BFCNT and BFCO prepared via a sol-gel method followed with a rapid thermal process (RTP) are studied, then the as-synthesized BFCNT and BFCO are introduced as an active layer into the silicon-based solar cell, after etching and texturing, we achieve a measured current density-voltage power conversion efficiency (PCE) of 3.9% with an introduction of TiO_2_ as the electron transport layer (ETL), NiO_*x*_:Cu as the hole transport layer (HTL), FTO and graphene as bottom and top electrodes, respectively. Then the adjustment of both polarization and magnetization on photovoltaic characteristics were studied, providing a valuable reference for the application of multiferroic materials and the design of novel photovoltaic devices.

## Results

**Fig. 1:**
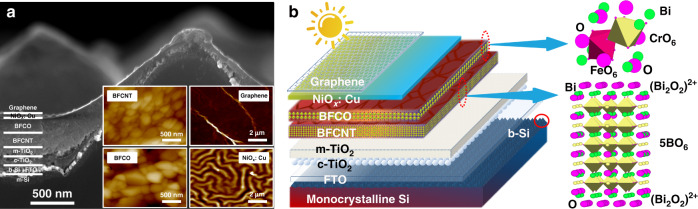
Microstructure and crystal texture. **a** SEM images of both the full device stack and the individual layers of BFCNT, BFCO, graphene, and NiO_*x*_: Cu. **b** Schematic of the multiferroic solar cells and the unit cell structures of both BFCNT and BFCO

The schematic, as well as SEM images of the multiferroic solar cell are exhibited in Fig. 1. When sunlight hits the absorption layer-BFCNT/BFCO heterojunction through the top graphene, the excited electrons and holes form bound states called excitons that break down under electric field, flow and transport respectively in ETL or HTL and finally arrive at the cathode and anode of the cell. The five corner-sharing BO_6_ octahedra-based perovskite-like layers are sandwiched by two fluorite-like (Bi_2_O_2_)^2+^ layers forming a typical half of an orthorhombic unit cell of BFCNT, the so-called AU structure^12^. However, for BFCO, it is more like an alternating arrangement of a FeO_6_ and a CrO_6_ octahedra layer along the [111] orientation^5^. XRD pattern of BFCNT/BFCO heterojunction, as well as the standard cards and the corresponding crystal structures of both BFCNT (ICDD No. 97-015-6257) and BFCO (ICDD No. 97-024-6426) are presented in Figure S1. The crystal grains of BFCNT pile layer after layer, forming a typical bismuth layer perovskite. And the BFCO grains usually gather together in the manner of a tetragonal structure. For the textured b-Si, pyramids shaped porous villi structure of ~108 nm thick covers on the surface of monocrystalline silicon, increasing the superficial area and therefore the ability of light trapping and absorption. The gauze-like transparent graphene electrode is an ultrathin graphene monoatomic layer, conducive to the pass-through of light and the transport of holes.

**Fig. 2:**
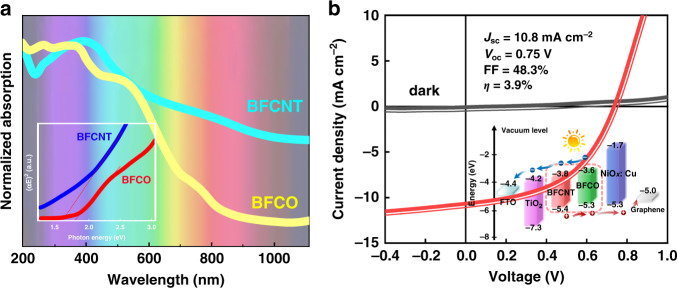
The optical and photovoltaic properties. **a** UV-Vis-NIR spectra of both BFCNT and BFCO, in arbitrary units (a.u.), the *inset* is the corresponding (αE)^2^ versus energy plots. **b** *J-V* characteristic under AM 1.5 G illumination of the solar cells. The *inset* shows the energy-level diagram based on UPS results showing the valence, Femi, and conduction energy of each component material

The optical characteristics of the as-grown BFCNT and BFCO films investigated by UV-Vis-NIR spectrophotometry measurements are presented in Fig. 2a. The films exhibit strong absorption in a large range from 200–800 nm. The bandgaps are evaluated to be 1.62 ± 0.04 eV and 1.74 ± 0.04 eV for BFCNT and BFCO films respectively by the corresponding *Tauc* plots, where the optical absorption coefficient (*α*) relates to bandgaps (*E*_*g*_) via *Planck’s* constant (*h*) and the frequency of the incident photon (*υ*) as: *α* = (*hυ−E*_*g*_)^1/2^ with *hυ* as the photon energy (*E*), consistent with the previous studies^3,5,6,12^.

Figure 2b presents the photovoltaic property of the b-Si solar cell based on multiferroic BFCNT/BFCO heterojunction. The dark *J-V* curve behaves like a rectifier diode with a dark current density of about 0.78 mA cm^−2^ at +1.0 V. Under the simulated AM 1.5 G illumination, we obtained the *V*_*oc*_, the short-circuit current density (*J*_*sc*_), and fill factor (*FF*) with 0.75 V, 10.8 mA cm^−2^, and 48.3%, respectively. This yields an energy conversion efficiency (PCE, *η*) of 3.9%. The comparison of the performance of our device with other reported is exhibited in Table S1. The normalized PV performance parameters (shown in Fig. S4) tested around 25 °C under humidity of ~30% indicated that such a multiferroic heterojunction black silicon solar cell presents a high stability.

To better understand the photovoltaic characteristics of the device and the effective transport of photo-generated carriers, the conduction and valence energies of each layer involved in the solar device are analyzed employing UPS. It is worth noting that only the UPS results based on ordered domain BFCNT and BFCO are qualitatively analyzed for the complexity of the coexistence of ordered/disordered phases in both BFCNT and BFCO films, making the construction of the energy band diagrams challenging. As illustrated in the *inset* of Fig. 2b, Fig. S2, and Table S2. that the band edge positions of TiO_2_, BFCNT, BFCO, and NiO_*x*_: Cu are well aligned, which is beneficial to the effective transport of photo-generated carriers.

**Fig. 3:**
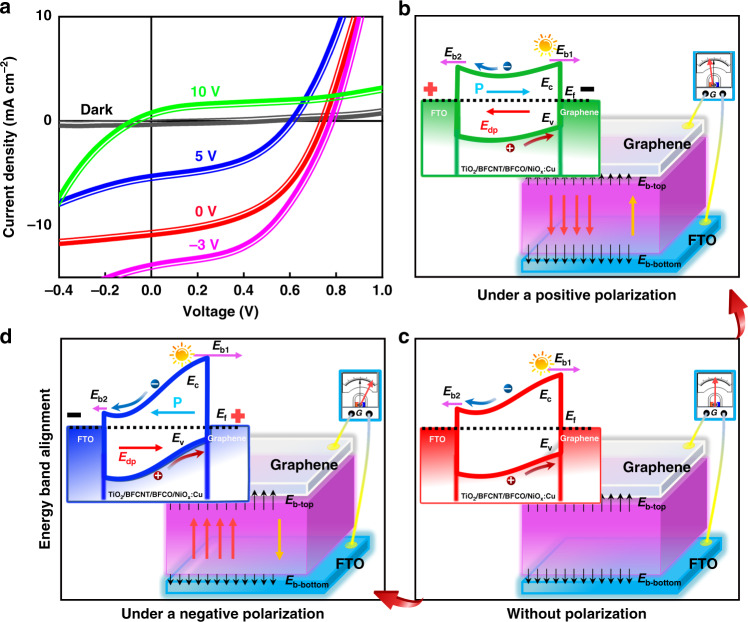
Modulation of polarization on photovoltaic properties. **a***J-V* characteristics at different polarizing voltages under AM 1.5 G illumination. **b**–**d** Schematic of both energy band alignments and the corresponding changes of the built-in electronic field without and with polarization

Ferroelectric polarization can regulate effectively the built-in electric field and the transport of charge carriers, thus causing a regulation for photovoltaic performances^28^. As shown in Fig. 3, leaving aside *Schottky* barriers between electrodes and the heterojunction, on one hand, a downward built-in depolarization electric field (*E*_*dp*_) originating from the deflection of dipoles appears while exerting a voltage of +5 V or more upon FTO, which hinders the migration rate of excitons, thus weakening the photovoltaic performance of the solar cells. Both *J*_*sc*_, *V*_*oc*_, and *FF* are smaller than that of the original performance without polarization, causing a relatively lower PCE according to the following equations^28^

1 $$\eta = \frac{{P_{\max }}}{{P_{in}}} = \frac{{V_{oc}J_{sc}FF}}{{P_{in}}}$$

where *P*_*in*_ is the product of solar cell area (*S*, m^2^) and light amplitude (*l*, w · m^−2^). In turn, a negative one (−3 V) promotes the migration rate of electron-hole pairs, improving the photovoltaic performance. On the other hand, this change is also due to the modulation of the energy band induced by polarization reversal. The accumulation of positive (negative) surface charges at the head (or tail) side of the polarization vector (as depicted in Fig. 3b, d) makes the energy levels of the active layer down (or up), resulting in a decrease (or increase) of the barrier height, which becomes large enough for positive poling to reverse the original band bending of the device structure. Apparently, switching the ferroelectric polarization will change and even reverse both *V*_*oc*_ and *J*_*sc*_ of the device, demonstrating that the ferroelectric polarization can modulate the transport of charge carriers and PV effects in our devices by tuning the energy band and the built-in depolarization electric field.

**Fig. 4:**
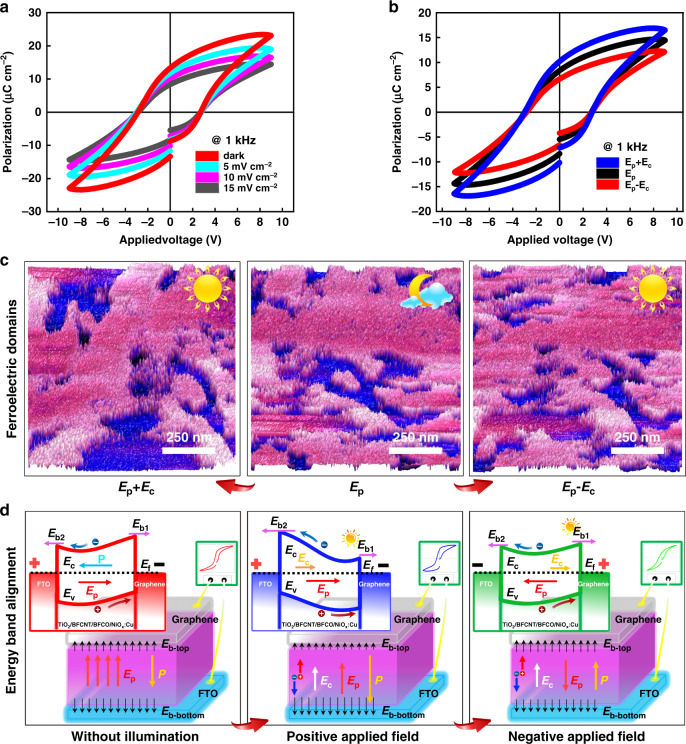
Effect of illumination on ferroelectric polarization. **a** Changes of P-V loop with different power densities of the light source. **b** Effect of illumination and field direction on ferroelectricity of BFCNT/BFCO heterojunction. **c** Evolution of ferroelectric domains under different conditions. **d** Schematic of both energy band alignment and the corresponding changes of the built-in polarization with and without illumination

In turn, illumination is also important for regulating the direction of dipoles, the evolution of ferroelectric domains, and the intensification of leakage current. As depicted in Fig. 4a, when a positive voltage was applied upon FTO, the ferroelectric polarization of BFCNT/BFCO heterojunction increases with the increasing of the power density of light sources under illumination. However in Fig. 4b and c, illumination changes the arrangement of ferroelectric domains, a positive voltage promotes the polarization and makes more ferroelectric dipoles redirect, but a negative one hinders it. It is known that the redirection of dipoles usually forms a ferroelectric polarization in the active layer under a steady electric field. However, when the light hits the active layer, a small built-in field (*E*_*c*_, upward) forms due to the shift and ballistic current induced by the non-central symmetrical electronic structure under the influence of back-to-back *Schottky* barriers of electrodes and polarization modulation of multiferroics (depicted in Fig. 4d)^29^. Once a positive voltage like +5 V or more was applied upon FTO, an upward polarization field (*E*_*p*_) appears in multiferroics and the energy band bends towards FTO, the final electric field intensity *E* = *E*_*p* _+ *E*_*c*_, causing more dipoles to redirect along the direction of the electric field and changing the arrangement of ferroelectric domains, which improves the ferroelectric polarization to some extent, otherwise, the energy band bends towards graphene and *E* = *E*_*p* _− *E*_*c*_, thus weakening the orientation of dipoles, thus changing the arrangement of ferroelectric domains and deteriorating the polarization. Evidently, illumination changes the arrangement of ferroelectric domains and the macroscopic polarization by the combination of the electric field caused by the directional movement of photo-generated carriers and the ferroelectric polarization electric field.

**Fig. 5:**
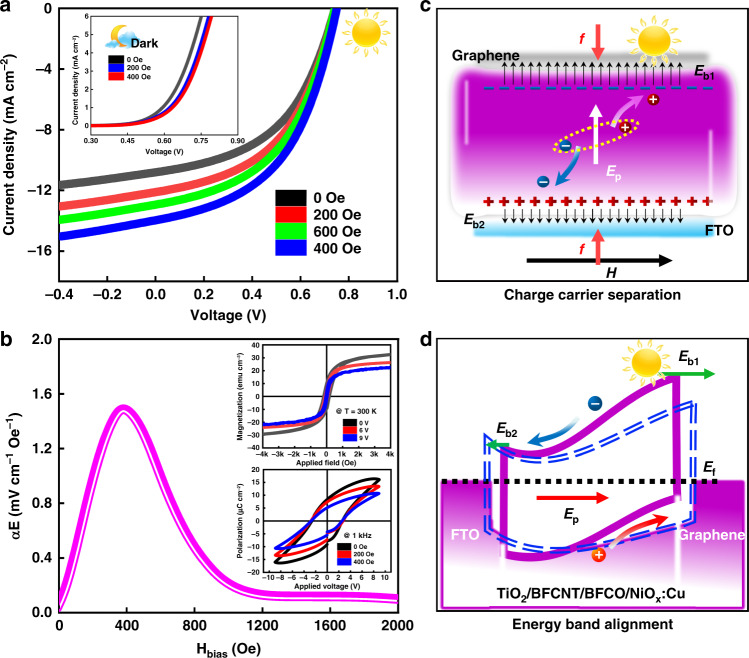
Modulation of magnetization on photovoltaic properties. **a***J-V* characteristics under different magnetic field intensities. **b** The magnetoelectric coupling coefficient (*α*_*E*_) along with the applied magnetic field, the *insets* show mutual regulations of electric and magnetic fields. **c** and **d** Schematic of both micro-deformation and energy band alignment under magnetization

The interaction between the magnetic field and light is the so-called magneto-optical effect, including the familiar *Faraday*, *Zeeman,* and *Kerr* magneto-optical effect, etc., inextricably linked to the magnetization of materials. Among dielectric materials, multiferroics have attracted much more attention for the coexistence of ferroelectric and ferromagnetic ordering, as well as interesting magnetoelectric coupling properties, providing a possibility for the regulation of the next-generation novel multifunctional devices, such as new-type solar cells, memristors, data storage units, *etc*. In multiferroic photovoltaic devices, the magnetoelectric coupling effect cannot be ignored in regulating the photovoltaic characteristics of the multiferroic solar cell as shown in Fig. 5b and Fig. S3. The detailed explanations are described in part 2 of the supplementary information. In brief, this phenomenon can be realized by the product of composite materials as follows^30^.

2 $$\alpha = \frac{H}{S} \times \frac{S}{P}$$

where *α* refers to the magnetoelectric coupling effect, *S* represents the strain, *H* and *P* are magnetic field and ferroelectric polarization, respectively. Once a magnetic field was applied perpendicular to the direction of illumination, the whole absorption layer will undergo a slight deformation due to the magnetostriction. Specifically, the absorption layer will stretch in the direction of the magnetic field, causing shrinkage to some extent along the direction of illumination. This stress will be transferred to the piezoelectric phase mainly through the phase interface due to the magnetoelectric coupling effect, resulting in a corresponding longitudinal deformation, which distorts the lattice to some extent although the deformation is reversible. This distortion will inevitably increase the carrier scattering probability (the scattering mainly originates from ionized impurities, lattice vibrations, lattice defects, intercarrier scattering, *etc*.), which will shorten the mean carrier-free time (*τ*) and reduce the mean carrier-free path (*λ*), ultimately leading to an enhancement of the carrier mobility (*μ*) because $\mu \propto \tau \propto \lambda \propto \frac{1}{P}$ ^31^, and a boosting of the solar cell performance. And meanwhile, a large number of positive charges will accumulate at the FTO electrode due to the piezoelectric effect (Fig. 5c)^32^, forming a polarization electric field (*E*_*p*_) from FTO to graphene. The final electric field intensity *E* = *E*_*b1* _+ *E*_*p* _− *E*_*b2*_, where the *Schottky* barrier voltage *E*_*b*1_ (Pt/multiferroics)>*E*_*b*2_ (multiferroics/FTO), shortening the energy band from FTO to graphene and elevates the barrier height of graphene(Fig. 5d). As a result, it promotes photo-generated electrons and holes moving towards the positive and negative electrodes. However, when the magnetostriction reaches the maximum (~400 Oe, shown in Fig. S3), the current density will not continue to increase but gradually decreases with further increase of the applied magnetic field as depicted in Fig. 5a, because when the external field exceeds 400 Oe, the activity of the magnetic domains of non-180° will be bound due to the attraction of the external magnetic field to the magnetic domain, which causes a decrease of magnetostrictive coefficient, thus decreasing the magnetoelectric coupling coefficient^33^.

In addition, it is nonnegligible that the *Zeeman* effect^34^ on the illumination of a multiferroic heterojunction photovoltaic device as described in Part 3 of the supplementary information. In short, the energy levels of the transition metal atoms (Fe, Ti, Co, Ni, and Cr) in the system will be polarized and spilt, which makes these impurity levels drifted away from the bandgap center, thus reducing the recombination rate of recombination centers to minority-carriers, prolonging the lifetime of minority-carriers and thus improving the efficiency of solar cells. It is worth noting that the splitting of the energy levels produced by these transition metal atoms is limited, they will not split indefinitely, and the splitting may produce many states deep within the bandgap that trap charge carriers and cause them to recombine non-radiatively, thus inducing local variations in photoluminescence and limiting the device performance as reported by Doherty, et al.^35^.

## Discussion

In summary, black silicon photovoltaic devices based on a multiferroic oxide BFCNT/BFCO heterojunction as well as a graphene layer are fabricated. The optical studies showed that the bandgaps of Aurivillius-typed BFCNT and double perovskite BFCO are 1.62 ± 0.04 eV and 1.74 ± 0.04 eV, which meets the requirements for active layers in solar cells. Under the simulated AM 1.5 G illumination, the *V*_*oc*_, *J*_*sc*_, *FF,* and *η* are 0.75 V, 10.8 mA cm^−2^, 48.3%, and 3.9%, respectively. The adjustments of an applied electric and magnetic field on the photovoltaic properties are investigated systematically, revealing that both magnetization and polarization of multiferroics can effectively tune the built-in electric field and the transport of charge carriers.

Last but not least, the mechanism of polarization and magnetization on regulating photovoltaic performances is rather complicated and requires further investigation. Nevertheless, the changes in photovoltaic property induced by an applied electric or magnetic field are evident from the experimental above, providing a new solution for the design of high-performance multiferroic oxide photovoltaic devices.

## Materials and methods

### Preparation of b-Si

A 180 ± 20 μm thick p-type double-sided polished monocrystalline Si (100) wafer with a resistivity of 0.5–1.5 Ω cm is selected for preparing b-Si. First, the clean Si wafer is pyramid-textured in an alkaline solution prepared by mixing 1.8% NaOH and 4.8% isopropanol alcohol with N_2_ bubbling^36^ for 40 min at 85 °C, then treated with HF solution (volume ratio of 1:50) for 5–10 min. Next, the nanotube-based textures were fabricated using a conventional metal-assisted chemical etching reaction^37^ in a mixed solution containing AgNO_3_ (0.02 M) and HF (4.5 M) at 85 °C. Residual Ag impurities are then removed from the nanoporous surface by immersing the substrate in an HNO_3_ (65%) solution.

### Precursor Synthesis

The compact TiO_2_ (c-TiO_2_) sol as a precursor of the ETL was prepared by the sol-gel method through hydrolysis and the aging of tetrabutyl titanate. Specifically, tetrabutyl titanate and concentrated hydrochloric acid (HCl) were dissolved in anhydrous ethanol separately and stirred adequately. The HCl solution was added into the tetrabutyl titanate solution and stirred adequately. After stirring at room temperature for 12 h and filtering to form a c-TiO_2_ sol. The mesoporous TiO_2_ (m-TiO_2_) used is brand dyesol with type 18NR-T shaped paste yellow. The mixture of TiO_2_ (18NR-T) and ethanol was stirred using a magnetic stirrer for 1 h at room temperature.

The BFCO solution (30 ml, 0.15 M) and BFCNT (50 ml, 0.03 M) as a precursor of the active layer were prepared by chelating the needed metallic nitrate with C_5_H_8_O_2_ in a mixture of C_2_H_4_O_2_ and appropriate C_3_H_8_O_2_ at 50 °C for 4 h. The solutions were then aged at room temperature for 48 h to get the corresponding precursors.

The NiO_*x*_: Cu sol as the precursor of HTL was obtained by dissolving 19:1 molar ratio of Ni(NO_3_)_2_·6H_2_O and Cu(NO_3_)_2_·3H_2_O in 2-methoxyethanol, the solution was stirred at 50 °C for 1 h, then the acetylacetone was added to the solution, then the mixed solution further stirred about 1 h at room temperature.

Finally, the graphene oxide (GO) solution as the precursor of graphene electrode of 150 mL, 0.5 mg mL^−1^ was reduced by hydrazine hydrate (the mass ratio of graphite oxide and hydrazine hydrate is 10:7–10:10) in a 90–95 °C water bath for 80–100 min. After filtering, the obtained solution was ultrasonically dispersed in deionized water.

### Device Fabrication

The c-TiO_2_ sol was first spin-coated onto the cleaned b-Si at 2000 r.p.m followed with annealing at 500 °C for 450 s in an RTP system, then m-TiO_2_ was coated on a c-TiO_2_ layer at 4000 r.p.m and annealed under the same conditions. After being treated with a 0.5 mL 1 M TiCl_4_ aqueous solution, the TiO_2_ layers were annealed again at 500 °C for 450 s to obtain the ETL. Next, BFCNT and BFCO layers were deposited successively at 3000 r.p.m followed by annealing at 500 °C for 450 s in air, forming the tandem active layer. The HTL was prepared by coating NiO_*x*_: Cu sol about 35 s at 3500 r.p.m and annealing at 250 °C for 450 s in air. In the end, the graphene solution was spin-coated at about 20 s at 2000 r.p.m. and drying at 150 °C under vacuum for 2 h to remove the moisture content^38^.

### Characterization

The phase structure information was determined by an X-ray diffractometer (XRD, SmartLab XG, Rigaku) with Cu K*α* monochromatic radiation (*λ* = 1.54 18 Å) at a scanning speed of 2° min^−1^ in steps of 0.02°. The microstructure was obtained via a field emission scanning electron microscope (SEM, Regulus 8100, Hitachi). The ferroelectricity of the active layer fabricated upon (100) oriented monocrystalline Si was analyzed by a ferroelectric test system (ferroelectric 200 V, Radiant Technologies) at 1 kHz, the light regulation of ferroelectric domains was investigated employing atomic force microscopy (AFM, Multimode 8, BRUKER) in modes of piezoelectric force microscopy (PFM) performed under a modulated sinusoid AC electrical field of 0.5 V with an SCM-PIT probe (Pt/Ir Coated Si Tips, 1–5 N m^−1^, 60–100 kHz) in a non-contact mode. The Femi energies and valence band edges of the materials were determined by ultraviolet photoelectron spectroscopy (UPS, Escalab 250Xi, Thermo Fisher). The optical measurements of the films were investigated by a UV spectrophotometer (U-4100, Hitachi) working in the ultraviolet-visible-near infrared (UV-Vis-NIR) range. The Current-Voltage measurements were performed using a solar simulator (96000, Newport-Stratfort 150 W) with simulated AM 1.5 spectrum and power density of 100 mW cm^−2^.

## Supplementary information

Multiferroic oxide BFCNT/BFCO heterojunction black silicon photovoltaic devices
