# Supplementary material for: Multiferroic oxide BFCNT/BFCO heterojunction black silicon photovoltaic devices
Source: Light Sci Appl. 2021 Sep 26;10:201. doi: 10.1038/s41377-021-00644-0 (PMC8473570; doi:10.1038/s41377-021-00644-0)
Supplement: Supplementary file 1 — Multiferroic oxide BFCNT/BFCO heterojunction black silicon photovoltaic devices [file 41377_2021_644_MOESM1_ESM.docx]

**Supporting Information**

**Multiferroic oxide BFCNT/BFCO heterojunction black silicon photovoltaic devices**

Kaixin Guo[[1]](#footnote-0), Xu Wang^1^, Rongfen Zhang^1^, Zhao Fu^2^, Liangyu Zhang^2^, Guobin Ma^1^,

and Chaoyong Deng^1^

* Author to whom correspondence should be addressed: cydeng@gzu.edu.cn.

**Table of contents**

1. **Preparation and characterization** ............................................................ 1
2. **Influence of magnetoelectric coupling on the performance** ................. 4
3. **Influence of the *Zeeman* effect on the performance** .............................. 6
4. **Supporting Tables** ..................................................................................... 9
5. **Supporting Figures** ................................................................................. 12
6. **Preparation and characterization**

**1.1 Preparation of b-Si**

A 180 ± 20 μm thick p-type double-sided polished monocrystalline Si (100) wafer with resistivity of 0.5~1.5 Ω·cm^-1^ is selected for preparing b-Si. First, the clean Si wafer is pyramid-textured in an alkaline solution prepared by mixing 1.8% NaOH and 4.8% isopropanol alcohol with N_2_ bubbling for 40 min at 85°C, then treated with HF solution (volume ratio of 1:50) for 5~10 min. Next, the nanotube-based textures were fabricated using a conventional metal-assisted chemical etching reaction in a mixed solution containing AgNO_3_ (0.02 M) and HF (4.5 M) at 85°C. Residual Ag impurities are then removed from the nanoporous surface by immersing the substrate in HNO_3_ (65%) solution.

**1.2 Precursor Synthesis**

The compact TiO_2_ (c-TiO_2_) sol as precursor of the ETL was prepared by the sol-gel method through the hydrolysis and the aging of tetrabutyl titanate. Specifically, tetrabutyl titanate and concentrated hydrochloric acid (HCl) were dissolved in anhydrous ethanol separately and stirred adequately. The HCl solution was added into the tetrabutyl titanate solution and stirred adequately. After stirring in room temperature for 12 h and filtering to form c-TiO_2_ sol. The mesoporous TiO_2_ (m-TiO_2_) used is brand dyesol with type 18NR-T shaped paste yellow. The mixture of TiO_2_ (18NR-T) and ethanol was stirred using a magnetic stirrer for 1 hour at room temperature.

The BFCO solution (30 ml, 0.15 M) and BFCNT (50 ml, 0.03 M) as precursor of active layer were prepared by chelating the needed metallic nitrate with C_5_H_8_O_2_ in a mixture of C_2_H_4_O_2_ and appropriate C_3_H_8_O_2_ at 50°C for 4 h. The solution were then aged at room temperature for 48h to get the corresponding precursors.

The NiO*_x_*:Cu sol as precursor of HTL was obtained by dissolving 19:1 molar ratio of Ni(NO_3_)_2_·6H_2_O and Cu(NO_3_)_2_·3H_2_O in 2-methoxyethanol, the solution was stirred at 50°C for 1 h, then the acetylacetone was added to the solution, then the mixed solution further stirred about 1 h at room temperature.

Finally, the graphene oxide (GO) solution as precursor of graphene electrode of 150 mL, 0.5mg·mL^-1^ was reduced by hydrazine hydrate (the mass ratio of graphite oxide and hydrazine hydrate is 10:7~10:10) in a 90~95°C water bath for 80~100 min. After filtering, the obtained solution was ultrasonic dispersed in deionized water.

**1.3 Device Fabrication**

The c-TiO_2_ sol was first spin-coated onto the cleaned b-Si at 2000 r.p.m followed with an annealing at 500°C for 450s in a RTP system, then m-TiO_2_ was coated on c-TiO_2_ layer at 4000 r.p.m and annealed under the same conditions. After treated with a 0.5mL 1M TiCl_4_ agueous solution, the TiO_2_ layers were annealed again at 500°C for 450s to obtain the ETL. Next, BFCNT and BFCO layers were deposited successively at 3000 r.p.m followed with an annealing at 500°C for 450s in air, forming the tandem active layer. The HTL was prepared by coating NiO*_x_*:Cu sol about 35s at 3500 r.p.m and annealing at 250°C for 450s in air. In the end, the graphene solution was spin-coated about 20s at 2000 r.p.m. and drying at 150°C under vacuum for 2h to remove the moisture content.

**1.4 Characterization**

The phase structure information was determined by an X-ray diffractometer (XRD, SmartLab XG, Rigaku) with Cu K*α* monochromatic radiation (*λ* = 1.54 18 Å) at a scanning speed of 2° min^−1^ in steps of 0.02°. The microstructure was obtained via a field emission scanning electron microscope (SEM, Regulus 8100, Hitachi). The ferroelectricity of the active layer fabricated upon (100) oriented monocrystalline Si was analyzed by a ferroelectric test system (ferroelectric 200V, Radiant Technologies) at 1 kHz, the light regulation of ferroelectric domains was investigated employing an atomic force microscopy (AFM, Multimode 8, BRUKER) in modes of piezoelectric force microscopy (PFM) performed under a modulated sinusoid AC electrical field of 0.5 V with a SCM-PIT probe (Pt/Ir Coated Si Tips, 1-5 N·m^−1^, 60-100 kHz) in a non-contact mode. The Femi energies and valence band edges of the materials were determined by ultraviolet photoelectron spectroscopy (UPS, Escalab 250Xi, Thermo Fisher). The optical measurements of the films were investigated by a UV spectrophotometer (U-4100, Hitachi) working in the ultraviolet-visible-near infrared (UV-Vis-NIR) range. The Current-Voltage measurements were performed using a solar simulator (96000, Newpret-Stratfort 150W) with simulated AM 1.5 spectrum and power density of 100 mW·cm^-2^.

1. **Influence of magnetoelectric coupling on the performance**

Multiferroic materials present simultaneous ferroelectric, ferromagnetic and magnetoelectric coupling[1,2]. Once a constant magnetic field is applied to multiferroic materials, the materials will stretch along the magnetic field direction due to the magnetostrictive effect, causing a shrinkage to some extent along the direction of illumination. This stress will be transferred to the piezoelectric phase mainly through the interface due to the magnetoelectric coupling effect, resulting in a corresponding longitudinal deformation, which distorts the lattice to some extent although the deformation is reversible[3-5]. This distortion will inevitably increase the carrier scattering probability (the scattering mainly originates from ionized impurities, lattice vibrations, lattice defects, intercarrier scattering, *etc*.), which will shorten the mean carrier free time (*τ*), reduce the mean carrier free path (*λ*), ultimately leading to an enhancement of the carrier mobility (*μ*) because $\mathrm{\mu}\propto\tau\propto\lambda\propto\frac{1}{P}$[6].

(1) $\tau=\frac{1}{P}$ (2) $\tau=\frac{\lambda}{\overline{\nu}}$ (3) $\mu=\frac{q}{m^{\text{*}}}\times\tau$

It is well known that elemental doping mechanisms, distortions and defects in materials can change the carrier mobility and the mean free path[6]. Our multiferroic heterojunction thin films contain a certain amount of transition elements (Fe, Co, Ni, Cr), and the introduction of these elements with different radii will cause distortions in the lattice, and increase the carrier scattering probability, thus leading to a larger carrier mobility.

However, when the magnetostriction reaches the maximum (~400 Oe), the current density will not continue to increase but gradually decreases with further increase of the applied magnetic field as depicted in Fig. 5a, because when the external field exceeds 400 Oe, the activity of the magnetic domains of non-180° will be bound due to the attraction of the external magnetic field to the magnetic domain, which causes a decrease of magnetostrictive coefficient, thus decreasing the magnetoelectric coupling coefficient[7].

**Reference:**

1. H. P. Zhou, Q. Chen, G. Li, S. Luo, T. B. Song, H. S. Duan, Z. R. Hong, J. B. You, Y. S. Liu, Y. Yang. Interface engineering of highly efficient perovskite solar cells. Science, 2014, 345, 542-546.
2. K. X. Guo, R. F. Zhang, M. Zhang, Y. L. Hu, S. Yang, C. Y. Deng. Voltage-regulated magnetization reversal in BNTFC/LSMO composite thin film, Appl. Surf. Sci., 2020, 509, 144823.
3. Uwe Rau, Thomas Kirchartz. Charge Carrier Collection and Contact Selectivity in Solar Cells, Adv. Mater., 2019, 6(20): 1900252.
4. Y. Zhang, W. J. Jie, P. Chen, W. W. Liu, J. H. Hao. Ferroelectric and Piezoelectric Effects on the Optical Process in Advanced Materials and Devices, Adv. Mater. 2018, 30, 1707007.
5. J. Hao, Y. Zhang, X. Wei. Electric-Induced Enhancement and Modulation of Upconversion Photoluminescence in Epitaxial BaTiO_3_:Yb/Er Thin Films, Angew. Chem., 2011, 50, 6876.
6. M. Born, K. Huang. Dynamical Theory of Crystal Lattices, Oxford University Press, Oxford, 1954.
7. C. H. Yang, Y. M. Wen, P. Li, L. X. Bian. Influence of bias magnetic field on magnetoelectric effect of magnetostrictive/elastic/piezoelectric laminated composite. Acta Physica Sinica, 2008, 57, 7292-7297.
8. **Influence of the *Zeeman* effect on the performance**

The *Zeeman* effect means that the the energy levels of the transition metal atoms (such as Fe, Ti, Co, and Ti) in multiferroics will split and polarize while placed in an applied magnetic field, which makes these levels be separated from the band gap center, thus reducing the recombination rate of minority carriers in recombination centers, and prolonging the life of minority-carriers and improving the efficiency of the photoelectrodes. In an atom, the vector sum of orbital and spin magnetic moments of electrons is just the total magnetic moment of atoms, rotating and moving along the magnetic field, the additional energy (*∆E*) is

$\Delta E=g\mu_{B}BM_{J}$ (1)

where *g* is the *Lande* factor, which represents the relationship between total magnetic moment and angular momentum, and determines the splitting of energy levels in the magnetic field, *B* and *μ_B_* are the intensity and moments of magnetic fields respectively, the magnetic quantum number *M_J_* is 2*J*+1 in total ( *J* is the Internal quantum number), which means that an energy level can be split into 2*J*+1 sub levels in an applied magnetic field. The transition from energy level *E*_1_ to *E*_2_ will produce a beam of light (*hυ*= *E*_1_-*E*_2_) under no magnetic field, However, the energy of the emission line becomes,

$h\upsilon=(E_{2}+\Delta E_{2})-(E_{1}+\Delta E_{1})=h\upsilon+(M_{2}g_{2}-M_{1}g_{1})\mu_{B}B$ (2)

due to the split of both *E*_1_ and *E*_2_ once a magnetic field was applied, thus making these impurity levels splitted away from the band gap center. According to the net recombination rate (*U*) of the compounding theory, when the capture interfaces (*σ*) of electrons and holes are equal,

$U=\frac{\sigma\nu_{t}N_{t}(p_{n}n_{n}-n_{i}^{2})}{n_{n}+p_{n}+2n_{i}ch(E_{t}-E_{i})/kT}$ (3)

where *ν_t_* is the average velocity of thermal motion of minority-carriers, *N_t_* represents the concentration of recombination centers not trapped by electrons, *p_n_* and *n_n_* are carrier concentration of holes and electrons in matrix materials of n-type semiconductor, *n_i_* refers to the intrinsic concentration of matrix materials, *E_i_* is the energy level of recombination centers caused by impurities or defects, and *E_t_* means the energy level of the central region of band gaps, as well as the lifetime (*τ_p_*) of the minority-carriers determined by *U* according to$\tau_{p}=\frac{\Delta p}{U}$.

Therefore, it can be concluded that an external magnetic field makes the energy levels of the transition metal atoms in multiferroics be separated from the bandgap center, which reduces the recombination rate of these impurity atoms to the minority carriers and increases the lifetime of minority carriers, thus improving the conversion efficiency of photovoltaic devices.

It is worth noting that the splitting of the energy levels produced by these transition metal atoms is limited, they will not split indefinitely, and the spritting may produce many states deep within the bandgap that trap charge carriers and cause them to recombine non-radiatively, thus inducing local variations in photoluminescence and limiting the device performance as reported by Doherty, *et al*.

**Reference:**

1. D. R. Herrick, Symmetry of the quadratic Zeeman effect for hydrogen, Phys. Rev. A, 26 (1982) 323-329.
2. H. W. Babcock, Zeeman effect in stellar spectra, Astrophys. J. 105 (1946) 105.
3. M. G. Mayani, T. W. Reenaas, Shockley-Read-Hall recombination in pre-filled and photo-filled intermediate band solar cells, Appl. Phys. Lett. 105 (2014) 073904.
4. G. J. Nott, P. C. Findlay, J. G. Crowder, C. R. Pidgeon, A. M. White, Direct determination of shockley-read-hall trap density in insb/inalsb detectors, J. Phys. Condens. Mat. 12 (2000) L731.
5. J. Chen, Z. Z. Jiang, J. J. Lu, Y. S. Liu, Y. Y. Zhu. Effects of split-level energy on optoelectronic transport in nanocrystalline silicon, Acta Physica Sinica, 2010, 59(12): 8862-8869.
6. Doherty, T. A. S. et al. Performance-limiting nanoscale trap clusters at grain junctions in halide perovskites. Nature, 2020, 580, 360-366.
7. **Supporting Tables**

**Table S1**. Comparation of our device with other reports.

| No. | Device structure | *J_sc_* (mA·cm^-2^) | *V_oc_* (V) | *FF* (%) | PCE (%) | Ref. |
| --- | --- | --- | --- | --- | --- | --- |
| 1 | Al/b-Si/Ag (Cu-catalyzed chemical etching + post-treatment process) | 36.67 | 0.64 | 80.66 | 18.88 | (1) |
| 2 | Al/b-Si/pn/Ag | 8.1 | 0.31 | 22.62 | 0.57 | (2) |
| 3 | b-Si/pn/ITO/BiFeO_3_/Ag | 25.84 | 0.29 | 24.55 | 1.85 | (2) |
| 4 | Glass/ITO/Bi_5_FeTi_3_O_15_ nanotubers+graphene powder (0,1,2) | 3.8, 15.8, 21.6 | 0.36, 0.72, 0.74 | 16, 44, 60 | 0.22, 4.97, 9.56 | (3) |
| 5 | FTO/c-TiO_2_(50 nm)/m-TiO_2_(400 nm)/ultrathin PbTiO_3_/MAPbI_3_/CNT | 23.47 | 0.93 | 75 | 16.37 | (4) |
| 6 | FTO/TNP (or TNT)/BiFeO_3_/Dye&electrolyte/Pt/FTO | 8.4 (8.5) | 0.73 (0.69) | 67.0 (59.3) | 4.1 (3.5) | (5) |
| 7 | ITO/TiO_2_/Na_0.5_Bi_0.5_FeO_3_/Spiro-OMeTAD/Au | 7.21×10^-3^ | 0.43 | 24 | 0.0075 | (7) |
| 8 | ITO/YbFeO_3_/Pt/MgO (or ZnO) | 21×10^-3^ (10×10^-3^) | 0.52 (0.46) | 28.6 (26.3) | 0.0031 (0.0012) | (7) |
| 9 | Pt/LuMnO_3_ (or YMnO_3_)/ITO | 0.52 (0.55) | 0.71 (0.66) | 30 (30) | 0.11 (0.11) | (8) |
| 10 | SrTiO_3_/SrRuO_3_/BiFeCrO_6_/ITO (*f* = 2, 8, 14 Hz) | 11.7, 10.8, 2.8 | 0.79, 0.65, 0.66 | 36, 29, 27 | 3.1, 2.1, 0.5 | (9) |
| 11 | 5wt%Nd-doped SrTiO_3_/BiFeCrO_6_/ITO ( *f* = 2+8+14 Hz) | 20.6 | 0.84 | 47 | 8.1 | (9) |
| 12 | 5wt%Nd-doped SrTiO_3_/BiFeCrO_6_/(NiO)/ITO | 3.3 (7.97) | 0.42 (0.53) | 56 (48) | 0.8 (2) | (10) |
| 13 | SrRuO_3_/BiFeCrO_6_/ITO | 13.8 | 0.52 | 38 | 2.65 | (11) |
| 14 | Commercial monocrystalline Si (Maxeon Gen III, SunPower Corp.) | *I_SC_* (6.11~6.18) A | 0.71~0.73 | 81.34~81.79 | 23.1~24.3 | (12) |
| 15 | Commercial SiN/Al/poly-Si/Ag (THS) | *I_SC_* (9.004~9.139) A | 0.635~0.649 | 79.9~80.01 | 18.6~19.3 | (13) |
| 16 | b-Si/FTO/c-TiO_2_/m-TiO_2_/BFCO/BFCNT/NiO*x*:Cu/Graphene | 10.8 | 0.75 | 48.3 | 3.9 | Our Device |

**Reference：**

1. P. Wang, S. Q. Xiao, R. Jia, H. C. Sun, X. W. Dai, G. Y. Su, K. Tao. 18.88%-efficient multi-crystalline silicon solar cells by combining Cu-catalyzed chemical etching and post-treatment process, Solar Energy, 2018, 169, 153-158.
2. M. Halbwax, T. Samet. Micro and Nano-structuration of Silicon by Femtoseeond Laser: Application to silicon photovoltaic cells fabrication, Thin Solid Films, 2008, 516(20): 6791-6795.
3. H. W. Zheng, X. Liang, Y. H. Yu, K. Wang. Bi_5_FeTi_3_O_15_ nanofibers/graphene nanocomposites as an effective counter electrode for dye-sensitized solar cells. Nanoscale Research Letters, 2017, 12(1): 18.
4. Yinglong Yang, Zhenghao Liu, Wai Kit Ng, Lihua Zhang, Hua Zhang, Xiangyue Meng, Yang Bai, Shuang Xiao, Teng Zhang, Chen Hu, Kam Sing Wong, Shihe Yang. An Ultrathin Ferroelectric Perovskite Oxide Layer for High-Performance Hole Transport Material Free Carbon Based Halide Perovskite Solar Cells, Advanced Functional Materials, 2018, 29(1), 1806506.
5. Ho-Yong Joo, Su Bong Hong, Hosang Lee, Ji Hoon Jeon. Ferroelectric BiFeO_3_-coated TiO_2_ electrodes for enhanced photovoltaic properties of dye-sensitized solar cells. Journal of the Korean Institute of Electrical and Electronic Material Engineers, 2013, 26(3):198-203.
6. X. Wu, Z. Wan, J. Qi, M. Wang, Ferroelectric photovoltaic properties of perovskite Na_0.5_Bi_0.5_FeO_3_-based solution-processed solar cells, Journal of Alloys and Compounds, 2018, 750, 959-964.
7. H. Han, D. Kim, K. Chu, J. Park, S. Y. Nam, S. Heo, C. H. Yang, H. M. Jang, Enhanced switchable ferroelectric photovoltaic effects in hexagonal ferrite thin films via strain engineering, ACS Applied Materials & Interfaces, 2018, 10, 1846-1853.
8. H. Han, S. Song, J. H. Lee, K. J. Kim, G. W. Kim, T. Park, H. M. Jang, Switchable photovoltaic effects in hexagonal manganite thin films having narrow band gaps, Chemistry Materials, 2015, 27, 7425-7432.
9. R. Nechache, C. Harnagea, S. Li, L. Cardenas, F. Rosei. Bandgap tuning of multiferroic oxide solar cells. Nature Photonics, 2015, 9, 61-67.
10. Wei Huang, Catalin Harnagea, Daniele Benetti, Mohamed Chaker, Federico Rosei, Riad Nechache. Mutiferroic Bi_2_FeCrO_6_ based p-i-n heterojunction photovoltaic devices. Journal of Materials Chemistry A, 2017, 510355-10364.
11. R. Nechache, W. Huang, S. Li, F. Rosei, Photovoltaic properties of Bi_2_FeCrO_6_ films epitaxially grown on (100)-oriented silicon substrates, Nanoscale, 2016, 8, 3237-3243.
12. <https://www.enf.com.cn/pv/cell-datasheet/1740?utm_source=ENF&utm_medium=material_profile&utm_campaign=enquiry_company_directory&utm_content=2085>
13. <https://www.enf.com.cn/pv/cell-datasheet/1944>

**Table S2**. UPS results of each layer of the device.

| No. | Materials | CBM (eV) | VBM (eV) |
| --- | --- | --- | --- |
| 1 | NiO*_x_*:Cu | -1.7 | -5.3 |
| 2 | BFCO | -3.6 | -5.3 |
| 3 | BFCNT | -3.8 | -5.4 |
| 4 | TiO_2_ | -4.2 | -7.3 |

1. **Supporting Figures**


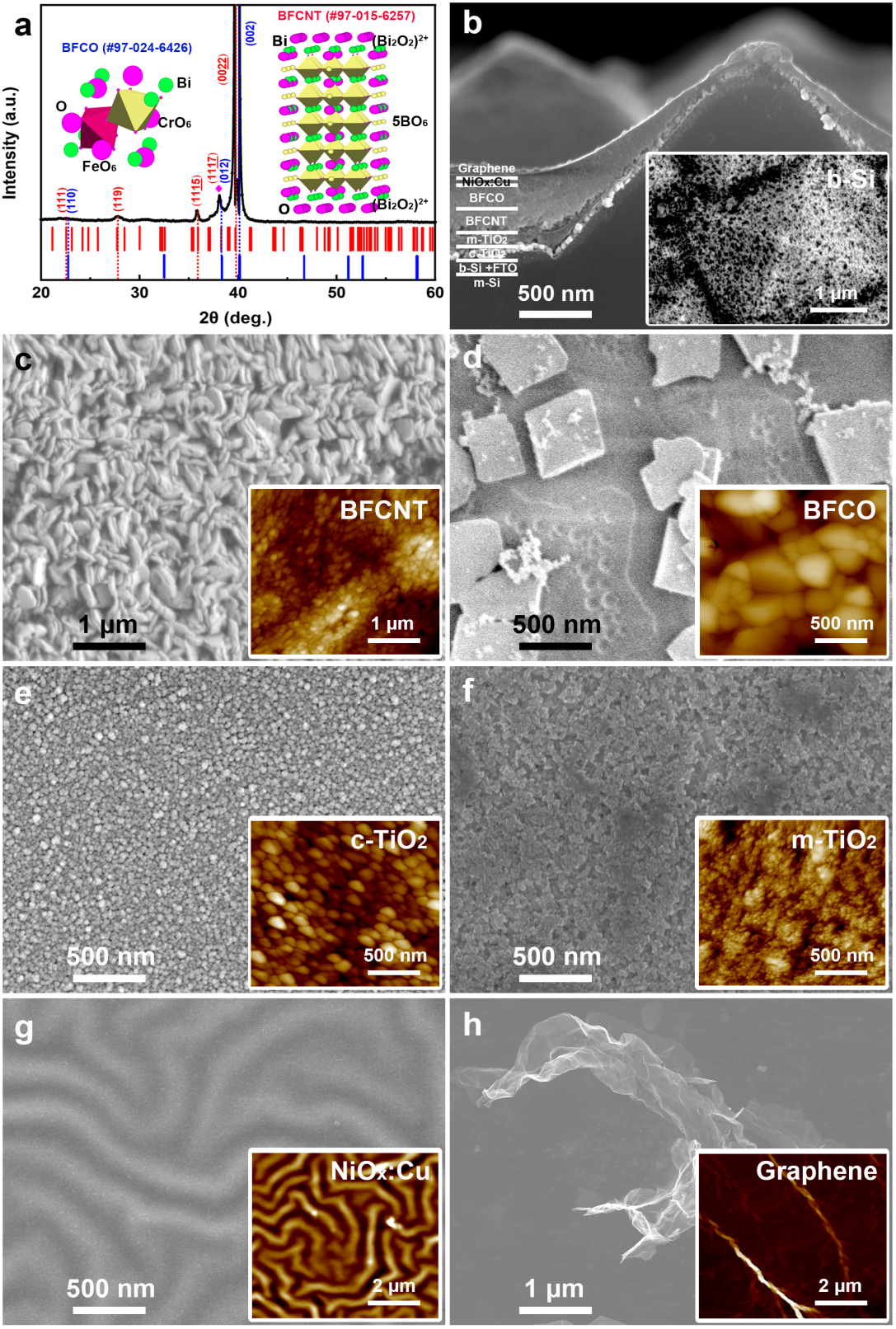


**Fig. S1**. **Phase composition and microstructure.** **a** XRD pattern of BFCO/BFCNT heterojunction. **b** Cross Section and surface morphology of black Si. **c**~**h** Surface morphology of each layer.


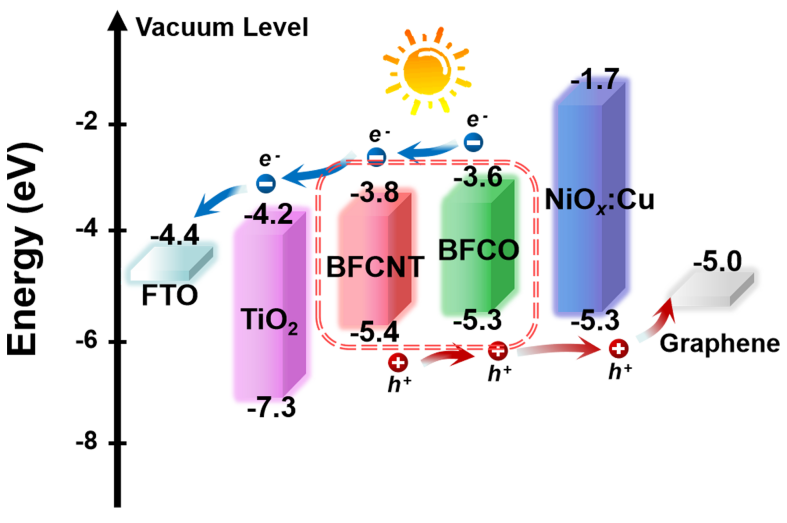


**Fig. S2**. **Energy-level diagram based on UPS results showing the valence, Femi and**

**conduction energy of each component material.**

**
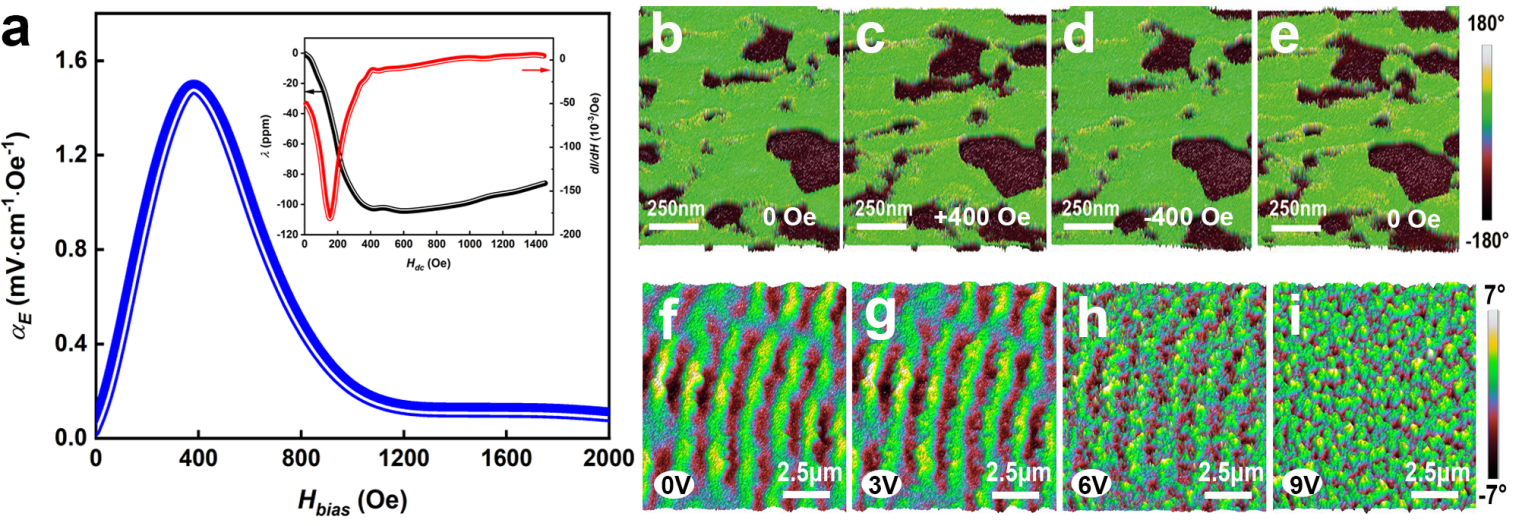
**

**Fig. S3**. **Magnetoelectric coupling of multiferroic BFCO/BFCNT heterojunction.** **a** Effect of both magnetostriction and magnetoelectric coupling, **b**~**e** Modulation of ferroelectric domains by different magnetism, **f**~**i** Tuning of ferromagnetic domains by different voltages.


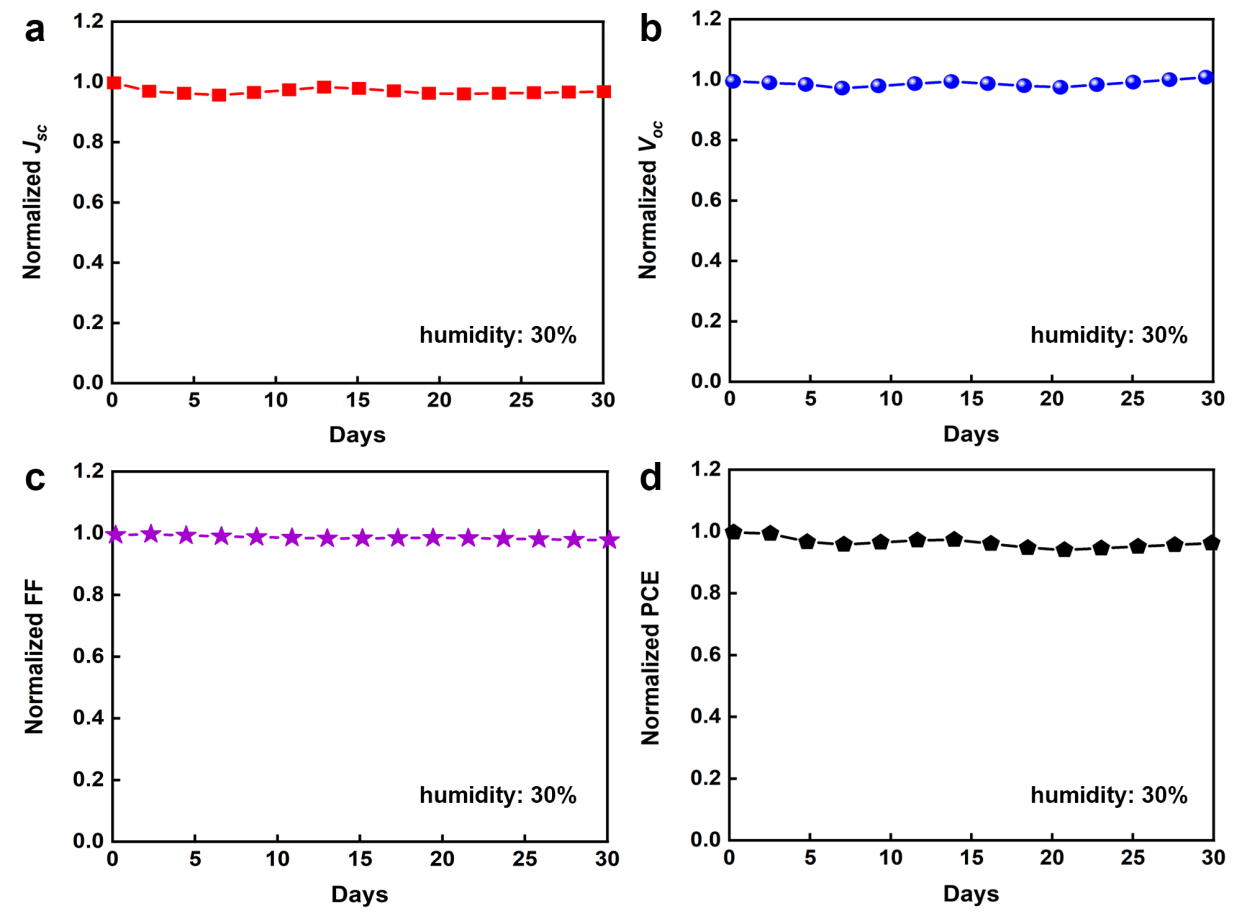


**Fig. S4**. **Stability test of devices stored in air without illumination under humidity of 30%.** The temperature was maintained around 25°C. Normalized PV performance parameters of **a** *Jsc*, **b** *VOC*, **c** *FF*, and **d** PCE.

1. Correspondence: Chaoyong Deng (cydeng@gzu.edu.cn)

   Key laboratory of Electronic Composites of Guizhou Province, College of Big Data and Information Engineering, Guizhou University, Guiyang 550025, Guizhou, China

   ^2^Guizhou College of Electronic Science and Technology, Guiyang 561113, Guizhou, China

   These authors contribute equal to this work: Kaixin Guo, Xu Wang [↑](#footnote-ref-0)
